# Quantification of functional hand grip using electromyography and inertial sensor-derived accelerations: clinical implications

**DOI:** 10.1186/1475-925X-13-161

**Published:** 2014-12-11

**Authors:** Jaime Martin-Martin, Antonio I Cuesta-Vargas

**Affiliations:** Departamento de Psiquiatría y Fisioterapia, Facultad de Ciencias de la Salud, Universidad de Malaga, Andalucia Tech, Instituto de Biomedicina de Malaga (IBIMA), Grupo de Clinimetria (AE-14), Malaga, Spain; School of Clinical Science, Faculty of Health Science, Queensland University Technology, Brisbane, Australia

**Keywords:** Assessment, Kinematic, Signal, Electromyography, Functions

## Abstract

**Background:**

Assessing hand injury is of great interest given the level of involvement of the hand with the environment. Knowing different assessment systems and their limitations generates new perspectives. The integration of digital systems (accelerometry and electromyography) as a tool to supplement functional assessment allows the clinician to know more about the motor component and its relation to movement. Therefore, the purpose of this study was the kinematic and electromyography analysis during functional hand movements.

**Method:**

Ten subjects carried out six functional movements (terminal pinch, termino-lateral pinch, tripod pinch, power grip, extension grip and ball grip). Muscle activity (hand and forearm) was measured in real time using electromyograms, acquired with the Mega ME 6000, whilst acceleration was measured using the AcceleGlove.

**Results:**

Electrical activity and acceleration variables were recorded simultaneously during the carrying out of the functional movements. The acceleration outcome variables were the modular vectors of each finger of the hand and the palm. In the electromyography, the main variables were normalized by the mean and by the maximum muscle activity of the thenar region, hypothenar, first interosseous dorsal, wrist flexors, carpal flexors and wrist extensors.

**Conclusions:**

Knowing muscle behavior allows the clinician to take a more direct approach in the treatment. Based on the results, the tripod grip shows greater kinetic activity and the middle finger is the most relevant in this regard. Ball grip involves most muscle activity, with the thenar region playing a fundamental role in hand activity.

**Clinical relevance:**

Relating muscle activation, movements, individual load and displacement offers the possibility to proceed with rehabilitation by individual component.

## Background

The hand is one of the fundamental elements of evolution for human beings and their interaction with the environment [1]. One element of great importance in the development of activities of daily living (ADLs) is the maintenance of sensory and motor skills (required for object manipulation) [2]. Changes in the functional capabilities of the hand could have a direct impact on the development of ADLs [3]. Therefore, the correct assessment of these skills is very important for planning rehabilitation processes [4]. One of the most complete tests of function is the Southampton Hand Assessment Procedure (SHAP) [5]. This is based on carrying out six fundamental tasks, namely power, ball and extension grip, and terminal, termino-lateral and tripod pinch. They are all performed in three modalities (light objects, heavy objects and interaction). The main outcome variable is the action time in each of the tasks [5].

However, the evaluation of the functional capabilities of any musculoskeletal structure – and hence the hand – should be more complete. It requires the use of instruments that are reliable and sensitive, allowing the analysis of muscle activity during contraction [6]. Surface electromyography allows the analysis of muscle activation through electrodes placed on the skin [7], a method used in previous pertinent studies [8–11], and the analysis of the function of the hand in healthy subjects based on the electrical activity of the muscles (electromyography) could provide reference values [8–11]. Furthermore, the use of inertial sensors (accelerometers) provides an overview of normal movement patterns [12] and inertial sensors are instruments that have shown high reliability and validity for the kinematic analysis of human movement [12] and own functional hand gestures [13, 14].

The analysis of the hand function based on electromyography and accelerometers with a gesture of reference has previously been undertaken [13, 14] to describe the maximum electromyography variables and the variation of acceleration in a task. Also, a combined electromyography and accelerometry system has been used by other authors to recognize movement [15]. However, no study has been found that performs an analysis of the function of the hand in which the electromyography signals and inertial sensors are integrated in the course of the six functional tasks in the SHAP. Linking both elements (electromyography and accelerometers) with the various functional tests of the hand could be the point of origin for a full functional assessment.

Therefore the objective of this study is to conduct a descriptive analysis of the six functional tasks of SHAP, integrating electromyographic and kinematic signals collected during the execution of each gesture. A secondary objective of this study is to compare the values of muscle activation and functional kinematics during the tasks included in the SHAP. Our hypothesis is that the kinematic and muscle activation records will be different for each of the functional gestures that compose the SHAP.

## Material and method

### Design

A descriptive study was carried out based on muscle activity and acceleration variables.

### Setting

This study was conducted at the Faculty of Health Sciences, University of Málaga. Data were collected during the months of January to March, 2014.

### Population

The sample used to conduct this study comprised 10 healthy subjects (five men and five women). The inclusion criteria were: aged between 18 and 35 years, right-handed, no skin disorders, the absence of any type of motor impairment in the upper extremity and the cognitive ability to understand the orders given. Exclusion criteria were left-handed (the glove used is adapted to the right hand, see material section), skin and upper extremity motor disorders, fractures and/or surgery of the right arm in the 12 months prior to the study, cognitive impairment that would prevent understanding of the tasks and implanted electrical medical devices (neurostimulator, pacemaker).

The participants were randomly selected and they had to be able to read, understand and sign the informed consent provided by the researcher. The informed consent showed the purpose of the study and the procedure to be followed, together with the questionnaires to be completed.

The study has the ethical support of the Ethics and Research Committee of the institution, in line with the Helsinki declaration [16]. In addition, the participant data have been treated in accordance with the Organic Law of Protection of Personal Data.

### Devices

#### Sample descriptive tools

The Upper Limb Functionality Index (ULFI) shows reliability values of 0.96 in the tests-retests, with a confidence interval of 95%, and the internal consistency based on Cronbach’s alpha is 0.89 in the Spanish version [17]. The psychometric properties of the QuickDASH questionnaire show a Cronbach’s alpha of 0.94 and a test-retest reliability of 0.95. The questionnaire consists of three parts: general (11 items), work (four items) and sports/music (four items) [18]. In addition, the dynamometer used was of a palmar pressure hydraulic type with a Jamar “Sammons Preston Rolyan” [19]. The palmar pressure force was obtained in kg/cm^2^ in two different positions: with the elbow in flexion and in extension. The Jamar hand dynamometer was adjusted based on the metacarpal measurements.

#### Kinematic device

Accelerometry was recorded with the Acceleglove [20] device, together with Acceleglove Visualizer recording software. The lycra AcceleGlove is fitted with six inertial accelerometer-type sensors: one on the back of the second phalanx of each finger and a sixth on the back of the hand. The sampling frequency of the device was 120 Hz. Each accelerometer records three spatial axes (X, Y, Z) from the AcceleGlove within a range of ±1.5 g. The unit of measurement of the device is “g”, (1 g = 9.8 m/s^2^), the value of universal gravitation. According to the manufacturer, the relationship between the axes of the glove is as follows: if the hand is horizontal, the Z axis is the gravity vector, which is perpendicular to the surface of the earth; the X (flexion and extension movements) and Y (adduction and abduction movements) axes are perpendicular to each other and to Z (ratio of movements). The hardware provides continuous acceleration values for each of the elements of the hand (fingers and palm). The reference unit for time measure is the time stamp of Unix 1 January 1970 [21].

The time in seconds was calculated by subtracting from the first time record and dividing by the sample rate. The magnitudes of the accelerations were calculated based on the vector module of each of the elements, . The time unit was transformed into seconds.

#### Electromyography device

The electromyography variables were registered using the MEGA ME6000 MT-M6T8-0-10 [22], measured in microvolts (μV), with the capture and data processing software Megawin 3.0.1, manufactured by Mega Electronics Ltd. (Kuopio, Finland). The sampling frequency of the device was 1000 Hz. Data capture was performed with ECG Lessa electrodes in two sizes [23]: child-size for the hand and adult-size for the forearm region. The electrodes were positioned and arranged in accordance with Perotto et al. [24] and the European Society of Electromyography (SENIAM) [25]. The skin was prepared to minimize the impedance experienced in electromyographic recording according to criteria established by SENIAM [25]. In addition, for this purpose, we excluded subjects whose BMI was equal to or greater than 35 kg/m 2 from the study.

### Procedure

Before starting the measurement protocol, anthropometric data of the subjects were recorded for descriptive analysis (Table 1). The description of the sample was completed using QuickDASH and ULFI questionnaires, as well as a test of hand dynamometry in two positions: with the elbow in flexion and in extension.

**Table 1:** **Description of the sample**

	Mean (SD)
Age (years)	**26.80** (±3.67)
Height (m)	**1.70** (±0.11)
Weight (kg)	**65.80** (±16.00)
Dynamo Max Ext (kg/cm^2^)	**38.60** (±13.07)
Dynamo Max Flex (kg/cm^2^)	**36.40** (±11.68)
ULFI (score)	**0.85** (±1.80)
QuickDash (score)	**4.31** (±10.30)
QuickDashWork (score)	**2.50** (±6.04)
QuickDash Sport (score)	**9.38** (±15.38)

The bold data is the mean value of each variable.

Subsequently, the AcceleGlove (outcomes recording device) and the electromyography electrodes were positioned. The muscles for which electrical activity was recorded were: the hypothenar muscles (opponens digiti minimi), the thenar muscles (flexor pollicis brevis), the first dorsal interosseous muscle, the flexors of the wrist (palmaris longus), the flexor carpi ulnaris muscle and the extensors of the wrist (extensor carpi radialis). To avoid a crosstalk effect in the electromyographic recording, the guidelines established by SENIAM [25] and Merletti [26] were followed. In addition, an exhaustive study of palpatory anatomy was conducted with each participant to improve electromyographic recording.

Tests of maximal voluntary muscle contraction (MVC) were performed [27] on each selected muscle. This test was performed three times for each muscle. The maximum recorded value of each muscle was considered the maximum value of activation and used as a reference for the normalization of muscle activation during the functional task.

**Figure 1:**
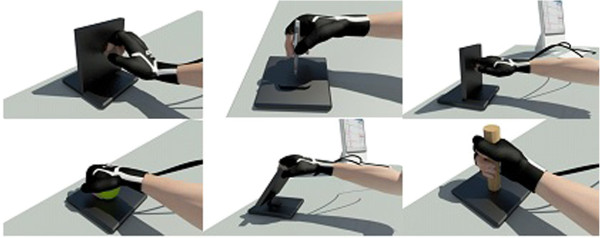
**Grip and pinch movement demonstration**

Once the recording of the maximum voluntary contraction was complete, the six functional gestures were recorded. Participants sat in a chair 50 cm high, with a straight back and the arm next to the body, the elbow flexed at 90°. An assessment table was placed opposite the participant on a 75 cm high surface on which the participant performed the following six functional movements (see Figure 1); three pinchs: *terminal pinch* (opposition between the thumb and index finger), *termino-lateral pinch* (opposition between the thumb and second phalanx of the index finger)*, three-tip pinch* (simultaneous opposition of the distal phalanx of the first three fingers, typical writing grip); three grips: *power grip* (simultaneous closing of all fingers to grasp a cylinder), *spherical grip* (simultaneous closing of all fingers to grasp a ball) and *extension grip* (opposition of the thumb from the rest of the fingers to grasp a closed book).

Participants placed the middle finger of the right hand on a reference mark located 20 cm from the interaction object. Participants received four acoustic alerts with five seconds of rest between them. The first alert was for the participants to remain at rest. The second alert was to move the hand to the interaction object and hold it. The third alert was to release the object and return to the starting point. The fourth alert was the end of the test. All participants performed each task three times. The second repetition of each functional movement was used for data analysis. The process was carried out with each of the functional movements. Electromyographic and kinematic data were recorded during movement and the rest time. Both devices were synchronized in real time and the data recorded simultaneously using DV Trigger (synchronization hardware), manufactured by Mega Electronics Ltd. (Kuopio, Finland). This element allows the recording process to be started simultaneously in two devices.

### Outcome variables

The outcome variables analysed in this study were of two different types: kinematic and electromyographic. The kinematic variables considered in this study were average acceleration of each finger and the palm of the hand (values for each of the functional gestures) and average acceleration of the gesture (values for each of the gestures made). Accelerometer values were measured in “g”, based on module vector of each sensor.

The electromyographic variables analysed in this study were mean muscle activation (the average muscle activation recorded for each muscle during the execution of the six functional gestures) and mean gesture activation (the average of all recorded muscle measurements analysed during the functional activation task). EMG values are presented in absolute values (uV) and normalized values (%). The normalized value represents the actual load or activation level required by a muscle with reference to the MVC value recorded. These variables provide an overview of each gesture.

### Data analysis

The quantitative outcome variables based on mean acceleration of the resultant vector and the surface electromyography values relative to MVC and absolute terms (microvolts) were extracted. Data were extracted by a blinded researcher with more than 10 years of experience in kinematic and electromyographic data analysis.

A descriptive analysis (mean and standard deviation) was carried out independently on the selected anthropometric data, questionnaires and dynamometric values. A descriptive analysis was then performed with the outcome variables. The software used was SPSS 15.0 [28] for Windows.

## Results

This study has 10 participants (five men and five women), with a mean age of 26.8 (±3.67) years old. The dynamometry tests performed did not reflect major changes in relation to the position of the arm; accordingly, no significant differences were observed in action between the two positions (elbow flexed at 90° and extended elbow). Furthermore, a description of the functional capacity of the upper limbs was undertaken; the questionnaires used were ULFI and QuickDash, with mean values of 0.85 (±1.80) and 4.31 (±10.30) respectively. The remaining descriptive variables for the sample can be observed in Table 1.

The mean value of acceleration is indicative of movement. It indicates the movement of each element in absolute value. This was calculated individually for each subject in each task in order subsequently to calculate an overall mean. Table 2 shows the average values of acceleration of each of the segments in all analysed gestures. The average acceleration of gestures ranged from 1.20 g (terminal pinch) to 1.28 g (tripod pinch). Also, the average acceleration of the segments ranged from 1.15 g (thumb) to 1.28 g (middle finger). However, the maximum acceleration record was 1.37 (±0.14)g (thumb – extension grip) and the minimum acceleration value recorded was 0.94 (±0.15) g (thumb – force grip). All the average acceleration values were recorded by segment for each of the gestures and the average acceleration of each gesture for each segment can be seen in Table 2.

**Table 2:** **Mean values of acceleration by vector and task (mean ± sd), in [g]**

	Thumb	Index	Middle	Ring	Pinky	Palm	MEAN
Tripod Pinch	**1.13** (±0.21)	**1.30** (±0.01)	**1.34** (±0.04)	**1.35** (±0.04)	**1.33** (±0.05)	**1.20** (±0.01)	**1.28**
Extension grip	**1.37** (±.014)	**1.26** (±0.02)	**1.25** (±0.03)	**1.25** (±0.02)	**1.26** (±0.02)	**1.21** (±0.04)	1.27
Spherical grip	**1.25** (±0.15)	**1.24** (±0.01)	**1.22** (±0.03)	**1.22** (±0.01)	**1.24** (±0.02)	**1.19** (±0.03)	1.23
Force grip	**0.94** (±0.15)	**1.26** (±0.02)	**1.34** (±0.01)	**1.31** (±0.03)	**1.33** (±0.06)	**1.24** (±0.01)	1.23
Lateral Pinch	**1.03** (±0.18)	**1.33** (±0.03)	**1.31** (±0.04)	**1.33** (±0.06)	**1.29** (±0.07)	**1.19** (±0.02)	1.25
Terminal Pinch	**1.18** (±0.117)	**1.23** (±0.02)	**1.22** (±0.03)	**1.19** (±0.02)	**1.16** (±0.03)	**1.23** (±0.03)	1.20
**MEAN**	1.15	1.27	**1.28**	1.27	1.27	1.21	

The bold data is the mean value of each variable.

The mean column bold data is the highest mean value of the set.

The absolute electromyographic values indicate the electrical activity recorded by a muscle in a given task (Table 3). The average value of the electrical activity in the tasks ranged from 40.20 μV (spherical grip) to 31.46 μV (lateral grip). The mean of the electrical activity of muscles ranged between 81.97 μV (thenar) and 18.78 μV (flexor wrist). However, the highest mean absolute value of the electromyography was 97.75 (±57.34) μV (thenar region – terminal pinch) and the mean minimum electromyographic value was 14.39 (±3.19)μV (flexor wrist – terminal pinch). Thus, the gestures with greater electrical activity were those with a spherical grip and the muscle with the highest record was the thenar region.

**Table 3:** **Mean values of muscle activity (mean ± sd) in [μV]**

	Hypothenar	Thenar	FirstDorsal	FlexWrist	FlexCarpi	ExtWrist	MEAN
Tripod pinch	**27.33** (±9.83)	**89.84** (±48.31)	**33.57** (±18.53)	**19.45** (±12.91)	**24.17** (±16.53)	**31.16** (±11.83)	37.58
Extension grip	**36.25** (±21.29)	**69.17** (±45.95)	**32.10** (±10.64)	**22.76** (±8.71)	**15.51** (±5.13)	**22.51** (±7.35)	33.05
Spherical grip	**47.04** (±17.78)	**93.75** (±70.33)	**24.60** (±11.89)	**21.70** (±11.60)	**27.09** (±14.21)	**27.04** (±11.01)	**40.20**
Force grip	**41.43** (±11.74)	**80.74** (±47.08)	**32.26** (±19.27)	**19.96** (±3.81)	**28.83** (±11.03)	**36.62** (±14.43)	39.97
Lateral grip	**19.43** (±7.15)	**60.55** (±47.81)	**46.06** (±25.96)	**14.42** (±2.15)	**18.90** (±2.68)	**29.43** (±10.92)	31.46
Terminal pinch	**18.59** (±5.79)	**97.75** (±57.34)	**30.55** (±25.37)	**14.39** (±3.19)	**16.08** (±4.89)	**50.35** (±27.05)	37.95
**MEAN**	31.67	**81.97**	33.19	18.78	21.76	32.85	

The bold data is the mean value of each variable.

The mean column bold data is the highest mean value of the set.

Electromyography normalized values can be seen in Table 4. According to normalized EMG, the maximum value possible is 100% MVC, i.e. maximal activation. In this regard, the muscles of the thenar region show greater activity relative to their capacity; the work load measurement (%MVC) is the highest, as can be seen in Table 4. On the other hand, spherical grip requires a greater contraction and shows the highest normalized average values.

**Figure 2:**
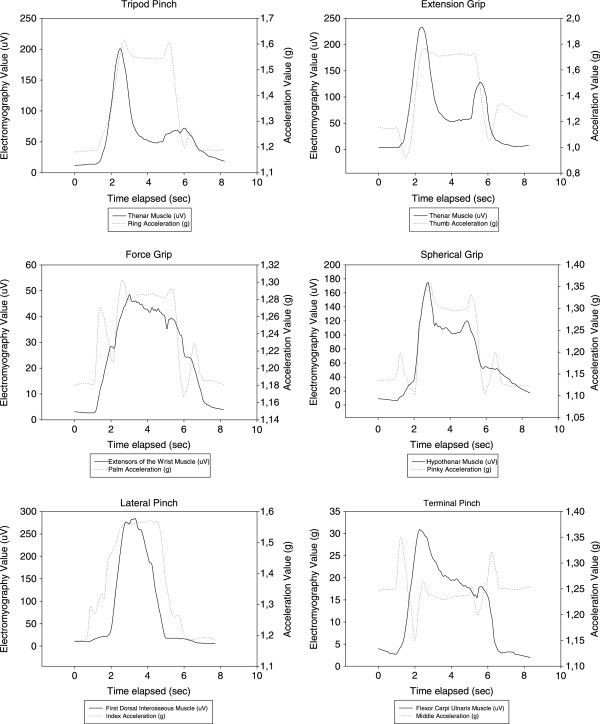
**Graphical representation of the tasks based on accelerometry [g] and electromyographyc [μV] values by subject.**

The temporal spectrum of a subject while performing the different tasks (tripod pinch, extension grip, force grip, spherical grip, lateral pinch and terminal pinch) based on sEMG variables of different muscles and the module vector of the ACC values throughout the time sequence was displayed on Figure 2. In order to obtain a more uniform curve in accordance with the following parameters: smoother negative exponential, sampling proportion 0.1, polynomial degree 1, minimal value x = 0 and maximal value x = 1 at 100th intervals.

**Table 4:** **Normalized mean values of maximum EMG activation (mean ± sd) in [%MVC]**

	Hypothenar	Thenar	FirstDorsal	FlexWrist	FlexCarpi	ExtWrist	MEAN
Tripod pinch	**3.49** (±2.28)	**6.09** (±1.86)	**3.22** (±2.38)	**1.22** (±0.61)	**6.60** (±7.04)	**3.22** (±1.69)	3.97
Extension grip	**4.11** (±2.25)	**4.38** (±1.94)	**3.69** (±2.30)	**1.57** (±0.58)	**3.19** (±1.85)	**1.98** (±0.93)	3.15
Spherical grip	**5.06** (±1.78)	**5.71** (±2.73)	**2.26** (±0.74)	**1.49** (±0.49)	**6.80** (±5.58)	**2.57** (±0.76)	**3.98**
Force grip	**4.68** (±1.58)	**5.13** (±1.53)	**3.00** (±1.51)	**1.45** (±0.43)	**6.35** (±3.71)	**3.15** (±0.61)	3.96
Lateral pinch	**2.13** (±0.51)	**4.53** (±4.09)	**4.67** (±2.73)	**0.98** (±0.25)	**3.66** (±1.19)	**2.39** (±0.80)	3.06
Terminal pinch	**2.18** (±0.89)	**6.48** (±2.80)	**3.23** (±2.24)	**1.04** (±0.44)	**3.10** (±1.01)	**4.09** (±2,93)	3.35
**MEAN**	3.60	**5.39**	3.34	1.29	4.95	2.90	

The bold data is the mean value of each variable.

The mean column bold data is the highest mean value of the set.

## Discussion

Addressing the primary objective of this study, the joint use of electrical activity and acceleration recording systems is a feasible way to analyse hand function, based on the SHAP tasks. This allows the comparison of the electromyographic and kinematic values during task performance. Both elements could be used to identify which functional tasks have greater acceleration based on the values of the hand segments when carrying out the tasks. Recording electrical activity by means of electromyography provides knowledge of muscle action in each movement.

On the basis of the results obtained, we demonstrate that muscle activation and acceleration of the segments of the hand change according to the task being performed. Therefore, in line with the hypothesis in this study, kinematic and muscle activation records will be different for each of the functional gestures that compose the SHAP.

The study reflects different patterns of movement and activation for each of the gestures. These patterns of muscle activation and movement (acceleration) can be used for functional recovery of the hand. The analysis of data derived from accelerometry and electromyography will allow the clinician to take a more direct approach in treatment, with understanding of which muscles should be recovered first, based on the level of activation and the elements with greater mobility.

### Kinematics

The mean acceleration values (Table 2) indicate that the tripod movement requires the greatest hand movement. This may be due to the placement which must be performed by the first three fingers of the hand in order to make the grip and the compensatory flexion carried out by the others. The middle finger is the hand element with the greatest acceleration. The highest mean values produced in the vector of the middle finger (1.28 g) should be treated with caution as being indicative of functionality (2 degrees of freedom). Individually, the thumb has a greater range of movement (3 degrees of freedom), despite registering lower acceleration values (1.15). The tripod grip pinch (1.28 g) has the highest average acceleration value, followed by the extension grip (1.27 g).

From a functional perspective, rehabilitation treatment should not be performed directly on the movement with greater mobility (in this study, the tripod grip) as this may hinder functional achievement. Subjects who are incapable of reaching the first set of goals may decide to drop out. In consequence, work on movements must be progressive and, based on the results of this study, start with the terminal pinch and ball grip, movements which produce less variation in the acceleration.

### Electromyography

There is a direct relationship between EMG absolute values (Table 3) and the normalized results (Table 4), as greater electrical activation implies greater individual load. However, there is a second relationship: force generation (and hence EMG values) is not the same for each muscle. In consequence, not all muscles can achieve the same absolute values; if they do, specific muscles may be subject to direct overload, as shown in Table 3. The higher muscle load values, absolute activity values (Table 3) and normalized values (Table 4) coincide in showing the importance of these elements. As mentioned above, it is not always the case that the muscles with greater electrical activity (Table 4) present the highest normalized values (Table 3). A single absolute activation value may present different normalized values depending on the muscle in question.

The muscle load record (Table 4) in healthy subjects shows how muscles behave with normal motor control and without alterations to activation. The muscles with the highest degree of activation in the series of six functional movements are the short flexor of the thumb (thenar region), followed by the flexor carpi ulnaris (Table 4); the ball grip requires the greatest muscle activity, whereas the lateral grip requires the least. This information can be used to guide the functional recovery process from least to greatest muscle activity.

### Kinematic and electromyography uses

Electromyography and accelerometry variables were analysed in a previous study [13, 14] that aimed to generate normal movement patterns by examining the changes in the different phases of a task. In addition, as in this study, electromyography (forearm) and accelerometry (forearm and wrist) have been used jointly by other authors for movement recognition. The results obtained could be used to estimate acceleration movements by means of electromyography values [8]. This shows the overall behaviour of both methods and their effectiveness for the analysis of movement, which can be used for prosthetic control. Gazzoni et al. [29] provide an analysis of the change in the activation and localization of muscle activity through electromyography according to the hand function and its angle. Furthermore, Ngeo et al. [30] use a simultaneous accelerometry and electromyography system for detecting muscle activation patterns. These patterns may be predictors of acceleration segments made by hand. The use of electromyography for the recognition of movement patterns has also been employed by Birdwell et al. [31]. In this case, the electromyographic signals provide the reference for virtual hand movements.

The forearm muscles selected for this study (flexor carpi ulnaris and palmaris longus) have been analysed by other authors to examine power grip at different intensities [9]. The results (Table 3 of this study) show that the flexor muscle with the greatest activity in mean values in power grip is the ulnar flexor carpi (28.83 μV ± 11.03), just as determined in the study carried out by Oskouei et al. [9].

Oskouei et al. [9] agree on the importance of the extensor muscles, although these are not assessed in their study; this aspect is analysed by Hoozemans et al. [10] as a predictor of strength (calibration procedure using fluctuating bursts of grip; the most effective predictions were between 27N and 47N), establishing the existence of a high relation between the extensor muscles, power grip and ability to counteract the wrist flexion. This factor is related to the importance of the wrist extensors, extensor carpi radialis (36.62 μV ± 14.43), as the forearm muscle with the most activity (Table 3).

A variable to take into account in rehabilitation treatment is muscle fatigue in repetitive tasks (three-tip pinch) analysed in twelve hand muscles [32]. In this regard, the intrinsic muscles of the thumb (abductor pollicis brevis and flexor pollicis brevis) show greater activity. In terms of activation, this is in line with the results of Table 4, which shows the thenar region (flexor pollicis brevis) as the most active in the hand in the three-tip grip (6.09%MVC ± 1.86).

Moreover, muscle stress changes according to the position adopted by the forearm to perform the different tasks, with the position of least fatigue for the forearm being neutral [33]. The neutral position is used in this study as a starting point in rest position and at the end of the task, with an approximate duration of two seconds in each period. However, the muscular load may be reduced through use of support surfaces. Onyebeke et al. [34] discuss the use of these elements of support while performing tasks of long duration with a mouse on a computer (similar to the pleota tennis grip), finding that these reduce muscle stress. The way in which the electrodes are arranged on the skin and their initial position may affect the electromyography results [11]. In this study, as in the results of Takala et al. [11], the electrodes were applied in the neutral position, after identifying the muscles by means of maximum voluntary contractions and in line with the indicated bibliography.

The angular opening of the wrist is one of the elements which most affects activation in the extensors digitorum, extensor carpi ulnaris and flexor carpi radialis [35]. These values are lower at high speed and the results may be affected; grip angle should be considered as a variable in studies related to the tasks.

## Conclusion

In consequence, based on the results obtained, the functional rehabilitation process should begin with the least demanding movements in terms of mobility (terminal pinch) and electrical load (lateral pinch). Individually, rehabilitation may start with the muscle with the greatest functional load, such as the short flexor of the thumb and the flexor carpi ulnaris. Based on mobility (accelerations), rehabilitation would start with the middle finger and ring finger. However, starting rehabilitation with the thumb should be considered, given its high functionality and integration in all hand movements.
